# Controllable protein phase separation and modular recruitment to form responsive membraneless organelles

**DOI:** 10.1038/s41467-018-05403-1

**Published:** 2018-07-30

**Authors:** Benjamin S. Schuster, Ellen H. Reed, Ranganath Parthasarathy, Craig N. Jahnke, Reese M. Caldwell, Jessica G. Bermudez, Holly Ramage, Matthew C. Good, Daniel A. Hammer

**Affiliations:** 1 Department of Bioengineering, University of Pennsylvania, 210 S. 33rd St, Philadelphia, PA 19104 USA; 2 Department of Chemical and Biomolecular Engineering, University of Pennsylvania, 220 S. 33rd St., Philadelphia, PA 19104 USA; 3 Department of Cell and Developmental Biology, University of Pennsylvania, 421 Curie Blvd., Philadelphia, PA 19104 USA; 4 Department of Microbiology, University of Pennsylvania, 3610 Hamilton Walk, Philadelphia, PA 19104 USA

## Abstract

Many intrinsically disordered proteins self-assemble into liquid droplets that function as membraneless organelles. Because of their biological importance and ability to colocalize molecules at high concentrations, these protein compartments represent a compelling target for bio-inspired materials engineering. Here we manipulated the intrinsically disordered, arginine/glycine-rich RGG domain from the P granule protein LAF-1 to generate synthetic membraneless organelles with controllable phase separation and cargo recruitment. First, we demonstrate enzymatically triggered droplet assembly and disassembly, whereby miscibility and RGG domain valency are tuned by protease activity. Second, we control droplet composition by selectively recruiting cargo molecules via protein interaction motifs. We then demonstrate protease-triggered controlled release of cargo. Droplet assembly and cargo recruitment are robust, occurring in cytoplasmic extracts and in living mammalian cells. This versatile system, which generates dynamic membraneless organelles with programmable phase behavior and composition, has important applications for compartmentalizing collections of proteins in engineered cells and protocells.

## Abstract (web summary)

Designer organelles with new biochemical functionalities are of great interest in synthetic biology and cellular engineering. Here the authors present a single-protein-based platform for generating synthetic membraneless compartments that is capable of enzymatically-triggered alterations to phase behavior and of recruiting and concentrating cargo proteins.

## Introduction

A subset of cellular compartments, such as the nucleolus and ribonucleoprotein (RNA-protein) granules, are membraneless organelles formed by liquid–liquid phase separation of intrinsically disordered proteins (IDPs)^1–3^. Biochemical and biophysical studies have begun to elucidate how these dynamic supramolecular assemblies of IDPs contribute to the mesoscale organization of the cytoplasm and nucleoplasm, and how they participate in such functions as spatiotemporal regulation of gene expression, signaling, and stress response^1–11^. The IDPs responsible for intracellular phase separation can often be expressed recombinantly and, above a critical concentration, spontaneously coacervate into protein-rich liquid droplets in equilibrium with a protein-poor phase^2,3,8,9^. We are interested in harnessing these IDPs to create bio-inspired materials—engineered membraneless compartments with novel functionality—that can be integrated into cells and protocells. Here we characterize a minimal, modular platform for engineering membraneless IDP-based compartments, enzymatically trigger liquid mixing and demixing, and demonstrate programmable cargo recruitment and release. As we illustrate, the material is a versatile platform for molecular engineering. Requiring only a single protein for phase separation into protein droplets, our platform exhibits phase behavior that can be logically gated, allows modular recruitment of multiple folded proteins, and forms dynamic organelles inside cells.

Engineering organelles to achieve new biochemical functionalities is an emerging field within synthetic biology^12–18^. One strategy is to modify endogenous organelles that are surrounded by membranes. Recent work in the field of metabolic engineering has demonstrated increased product titer by targeting metabolic pathways to endogenous organelles, such as peroxisomes^12–14^. The rationale is that colocalization and increased concentration of enzymes and substrates boosts reaction rates^14,19^. A second strategy is to design and express synthetic organelles in cells^15^. However, a significant obstacle to engineering both endogenous and synthetic membrane-bound organelles is controlling permeability and transport through membranes, which requires the inclusion of channels and receptors^14,20,21^. Bottom-up engineering of membrane-enclosed organelles is also inherently difficult because lipid biogenesis is complex and difficult to rewire. Membraneless organelles would provide for facile transport of substrates without the need for reconstitution. Additionally, although synthetic scaffolding molecules have been employed successfully to colocalize metabolic enzymes^16,22^, they, unlike organelles, cannot limit permeability and exclude inhibitors.

Liquid-phase condensation of IDPs offers a strategy to construct synthetic, genetically encoded, membraneless organelles that selectively encapsulate cargo proteins. Importantly, it is believed that membraneless organelles can fulfill many of the roles of their membrane-bound counterparts, such as functioning as intracellular chemical reactors^1,7^. However, the field currently lacks a platform by which synthetic organelle assembly, disassembly, and targeted cargo recruitment can be rapidly switched through specific external triggers. Synthetic proteins of low sequence complexity have been designed with programmable phase behavior and multiscale architecture, but lacking inducible mechanisms of assembly, disassembly, and cargo recruitment^23,24^. Recent research elucidated cargo partitioning into droplets formed from interacting pairs of multivalent proteins or proteins and RNA^25,26^, but for ease of engineering a synthetic organelle, we sought a minimal system comprised of only one protein component that phase separates with low critical concentration and in the absence of RNA. Although recent work demonstrated protein phase separation in response to blue light, continuous illumination was required to maintain phase separation^27^. Finally, phosphorylation/dephosphorylation offers a site-specific mechanism for triggering phase transitions, as shown recently for clustering of T-cell signaling molecules^28^ and complex coacervation of cationic peptides with RNA^29^, however these are multi-component systems and less suitable for bioengineering.

**Fig. 1:**
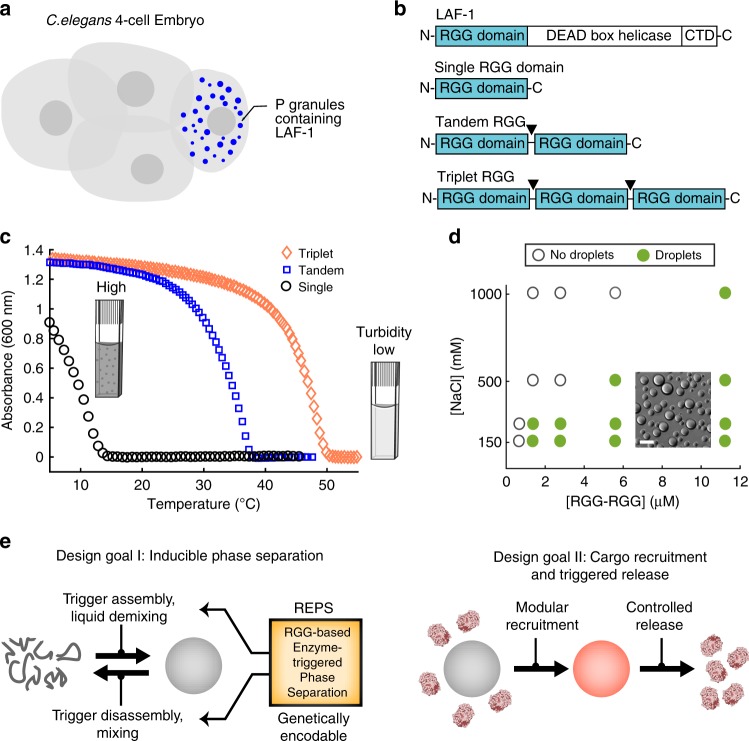
Phase separation of RGG-based IDP constructs. **a** Illustration of four-cell *C. elegans* embryo. LAF-1 is present in P granules, which contribute to germline specification. **b** Domain organization of LAF-1, RGG, RGG-RGG, and RGG-RGG-RGG. **c** Representative turbidity measurements show temperature-dependent phase behavior of RGG, RGG-RGG, and RGG-RGG-RGG at protein concentrations of 0.2 mg/mL (approximately 12 μM RGG domain concentration) in 150 mM NaCl buffer, pH 7.5. **d** Phase diagram of RGG-RGG as a function of salt and protein concentrations. Green markers indicate conditions at which optically resolvable droplets were observed. Inset: microscopy image of phase-separated RGG-RGG protein droplets. Scale bar: 10 µm. **e** Schematic illustrating design goals (i) inducible phase separation and (ii) cargo recruitment and triggered release

In this report, we characterize the material properties of a family of phase-separating proteins, which we manipulate to engineer bio-inspired, protein-based organelles. As a starting point for engineering protein phase behavior, we selected the RGG domain from LAF-1. LAF-1 is a member of the DDX3 family of RNA helicases and is found in P granules, which are membraneless organelles involved in specification of the germline in *Caenorhabditis elegans* (Fig. 1a). LAF-1 is comprised of an intrinsically disordered RGG domain at its N terminus, followed by a helicase domain and disordered C-terminal domain (Fig. 1b). Recent work revealed that the RGG domain is necessary to promote LAF-1 phase separation and itself can undergo liquid demixing^2^. The RGG domain is a low-complexity sequence, 168 residues in length, featuring 7 occurrences of the arginine-glycine-glycine sequence motif. It is especially enriched in glycine (approximately 35% of residues), followed by arginine (14%), asparagine (14%), aspartic acid (10%), serine (7%), and tyrosine (7%). IDP assembly into droplets is driven by weak, noncovalent interactions, and is therefore sensitive to salt concentration and temperature^2,23^. The LAF-1 RGG domain is representative of a class of IDPs that have an upper-critical solution temperature (UCST), remaining soluble at high temperatures and condensing out of solution upon cooling below a critical temperature^11,23^.

Here we design a modular, robust platform to generate synthetic membraneless compartments capable of enzymatically triggered alterations to phase behavior and of recruiting and concentrating cargo proteins. We term these RGG-based, enzyme-triggered, phase-separating systems, or REPS. By multimerizing the RGG domain from LAF-1 (RGG-RGG and RGG-RGG-RGG; illustrated in Fig. 1b), we find that proteins comprised of multiple RGG domains in series more readily self-assemble into liquid droplets than do ones containing a single RGG. Next, we engineer protease cut sites and protein-interaction motifs within this RGG-based scaffold, to switch phase behavior under physiological conditions. These liquid droplets exhibit limited permeability and size-dependent partitioning of exogenous molecules, but we leverage protein interaction motifs to selectively recruit cargo proteins that are normally excluded from RGG-RGG droplets. Further, we demonstrate controlled release of multiple cargos, in series or in parallel, by protease treatment. Importantly, the phase behavior and activities of our engineered material are retained in protocells and in living cells. These RGG-based materials, REPS, offer a generalizable, modular strategy to construct and control the behavior of synthetic membraneless organelles.

## Results

### Tandem RGG as a scaffold for engineering protein condensates

The RGG domain is necessary and sufficient for phase separation of LAF-1, yet on its own the RGG domain only weakly phase separates^2^, requiring non-physiological protein concentrations or low temperatures to demix into liquid droplets. Seeking a more robust platform for engineering membraneless organelles, we hypothesized that multiple RGG domains in tandem would more readily phase separate than a single RGG domain. Previously, increased valency in the SH3-PRM interaction pair has been shown to drive more robust liquid–liquid demixing^26,30,31^. Using recombinant methods, we expressed and purified multimeric versions of the RGG domain: a tandem RGG protein in which two RGG domains are connected by a short linker (RGG-RGG), and a corresponding triplet RGG protein (RGG-RGG-RGG). For comparison, we also expressed and purified a single RGG domain (schematic, Fig. 1b).

To compare the phase behavior of single, tandem, and triplet RGG, we used a spectrophotometric assay that measures temperature-dependent turbidity (Fig. 1c). The proteins were assayed in physiological buffer (150 mM NaCl, pH 7.5), with RGG domain concentration matched for all three constructs (at 12 µM RGG domain concentration). All three protein solutions exhibited UCST phase behavior—they were transparent at elevated temperatures, but upon cooling they phase separated and become visibly turbid (Supplementary Fig. 1A)—but their transition temperatures differed significantly depending on the number of RGG repeats. RGG was miscible at room temperature and phase separated only when the temperature was reduced below 15 °C. RGG-RGG phase separated at room temperature and even at physiological temperatures for tissue culture (Fig. 1c). It was miscible only at temperatures above approximately 40 °C. RGG-RGG-RGG phase separated even more readily, becoming turbid at temperatures below approximately 50 °C.

The temperature-dependent phase behavior of RGG-RGG can be cycled; turbidity appeared, disappeared, and then reappeared upon cooling, heating, and recooling (Supplementary Fig. 1B). The turbidity measured in the spectrophotometric assay arises from micrometer-sized droplets that are visible by light microscopy (Fig. 1d, inset). To further characterize the phase boundary of RGG-RGG, we used light microscopy to map droplet formation as a function of salt and protein concentrations at a fixed temperature of 25 °C (Fig. 1d). At 150 mM NaCl, RGG-RGG phase separated at protein concentrations >1 µM, comparable to the critical concentration necessary for the phase separation of full-length LAF-1^2^. Higher RGG-RGG concentrations favored droplet assembly, whereas higher NaCl concentrations promoted miscibility. These effects were confirmed using the spectrophotometric assay (Supplementary Fig. 1C and 1D). Microscopy showed that RGG-RGG droplets have dynamic, liquid-like properties; droplets rapidly fused and adopted spherical shape to minimize surface area (Supplementary Fig. 1E). From fluorescence recovery after photobleaching (FRAP) of labeled RGG-RGG droplets, we estimated a diffusion coefficient of *D* ≈ 0.085 µm^2^/s (Supplementary Fig. 1F), whereas LAF-1 in the absence of RNA was reported^2^ to have *D* ≈ 0.010 µm^2^/s. Similar to tandem RGG, triplet RGG protein exhibited phase behavior dependent on protein and salt concentration and formed spherical droplets that rapidly fuse (Supplementary Fig. 1G–I). Thus, proteins with multiple RGG domains in series phase separate more readily compared to single RGG. Furthermore, these proteins assemble into spherical liquid droplets in the absence of RNA in vitro under physiological conditions. Based on these favorable properties, we envisioned using multivalent RGG as a platform to implement two distinct design goals: (i) inducible phase separation through triggered droplet assembly and disassembly, and (ii) targeted cargo recruitment and triggered release (schematic, Fig. 1e).

### Reversing phase separation by triggered reduction in valency

**Fig. 2:**
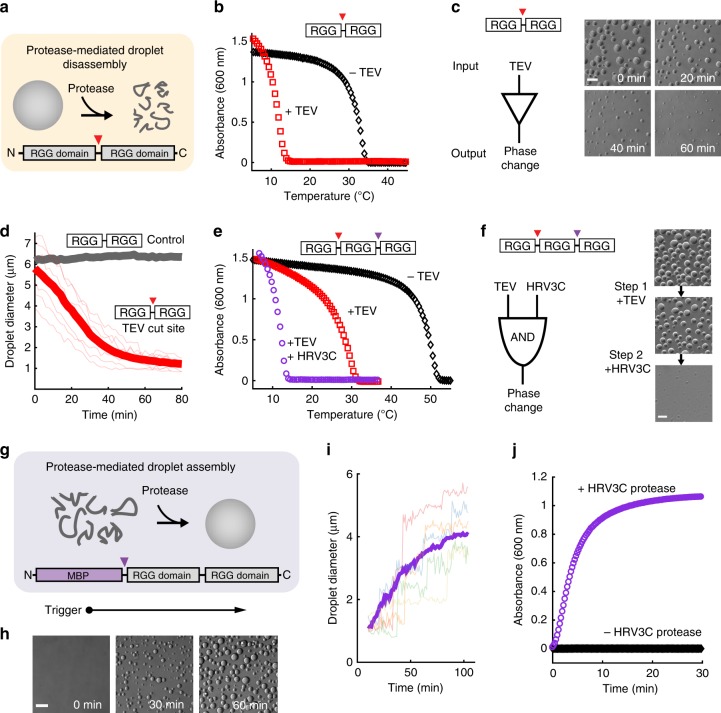
Protease-triggered assembly and disassembly of RGG-based protein droplets. **a** Schematic of protease-mediated disassembly of RGG-RGG droplets through reduction in valency. **b** Compared to untreated, the phase transition temperature of TEV protease-treated RGG-x-RGG (x = TEV cut site) is markedly reduced, matching that of single RGG. **c** Microscopy of TEV-triggered droplet dissolution. Time points 0, 20, 40, and 60 min after TEV addition (150 nM). Scale bar: 10 µm. **d** Analysis of droplet dissolution from time-lapse imaging: TEV cleavage rapidly reverses phase separation of RGG-x-RGG. Droplets formed by RGG-RGG lacking a cut site remain intact after adding TEV. TEV concentration: 150 nM. Representative individual traces (thin red lines) and population average (thick red line) for RGG-x-RGG, compared to population average for RGG-RGG droplets (thick gray line). Averages are of >750 droplets. **e** Protease treatment alters phase behavior of RGG-x-RGG-y-RGG (x = TEV protease cut site, y = HRV3C protease cut site). Treatment with TEV reduces phase transition temperature, however protein remains phase separated at room temperature. Upon treatment with both TEV and HRV3C, phase separation requires temperatures < 15 °C, consistent with the phase behavior of single RGG domain. **f** AND gate regulatory behavior of RGG-x-RGG-y-RGG revealed by optical microscopy. Upon treatment with TEV only, protein compartments remain intact and phase separated. Upon TEV treatment and addition of HRV3C protease, phase separation is reversed and droplets dissolve. TEV and HRV3C protease concentrations: 0.5 µM. Scale bar: 10 µm. **g** Schematic of protease-mediated droplet assembly by removal of a solubility-enhancing tag, MBP, from MBP-RGG-RGG. **h** Droplet assembly triggered by HRV3C protease monitored by time-lapse microscopy. HRV3C protease: 1 µM. Scale bar: 10 µm. **i** Analysis of droplet assembly from time-lapse videos. Traces of five representative individual droplets, along with population average (thick purple line) of >200 droplets. **j** Spectrophotometric assay of the kinetics of droplet assembly. Addition of HRV3C protease at *t* = 0 causes droplet assembly and a rapid increase in turbidity; the control, lacking protease, shows no increase in turbidity. For all experiments, initial concentrations of RGG-based constructs were 6 μM

Based on the enhanced phase behavior of tandem RGG, we devised a strategy for triggering droplet disassembly. We hypothesized that under physiological conditions, proteolytic cleavage of RGG-RGG into single RGG domains would reverse the phase behavior to that of single RGG, thereby causing droplet dissolution (schematic, Fig. 2a). We placed the Glu-Asn-Leu-Tyr-Phe-Gln-Gly recognition sequence, which is the target of tobacco etch virus (TEV) NIa protease, between the two RGG domains in RGG-RGG (termed RGG-x-RGG). TEV protease is well suited for cell engineering because it is highly specific, has no endogeneous targets in the mammalian proteome, and can be overexpressed in mammalian cells^32,33^. Protease activity cleaved nearly 100% of RGG-x-RGG into fragments of the appropriate molecular weight (Supplementary Fig. 2A). TEV cleavage of RGG-x-RGG is predicted to occur immediately downstream of the Gln in the TEV recognition site, yielding N- and C- terminal fragments with molecular weights of 17,843 and 17,907 Da, respectively. Using matrix-assisted laser desorption/ionization time-of-flight (MALDI-TOF) mass spectrometry, we observed prominent peaks at 17,844 and 17,909 Da (Supplementary Fig. 2B), in excellent agreement with the prediction. TEV did not cleave RGG-RGG lacking a cut site (Supplementary Fig. 2C), indicating the protease’s specificity.

TEV protease treatment of RGG-x-RGG altered the protein’s phase behavior, causing significant reduction in turbidity under physiological conditions (Fig. 2b and Supplementary Fig. 2D). This transformation could also be monitored by light microscopy, revealing time-dependent dissolution of the droplets (Fig. 2c). Protease activity therefore enables logic-based control of phase transitions. Image analysis revealed that the time required to disassemble 50% of droplet diameter was <30 min (Fig. 2d). RGG-RGG lacking the TEV recognition sequence exhibited no droplet disassembly, even after >1 h of treatment with TEV. This suggested that the reversal of phase separation in RGG-x-RGG was due to specific cleavage, which converted RGG dimers into monomers. To demonstrate the versatility of this droplet dissolution strategy, we also tested an RGG-RGG variant in which we placed a canonical thrombin recognition and cleavage site, Leu-Val-Pro-Arg-Gly-Ser^32,34^, between the two RGG domains. Thrombin treatment resulted in conversion of tandem into single RGG, reduction of turbidity at room temperature, and droplet dissolution (Supplementary Fig. 3). Thus, the insertion of protease recognition sequences between the RGG domains is modular and can be extended to different protease-cleavable motifs.

To test whether we could achieve further logic-based control of protein phase behavior, we designed a form of triplet RGG containing additional control points for programmable disassembly. We inserted two different protease cleavage sites into triplet RGG, resulting in RGG-x-RGG-y-RGG, where x denotes the recognition sequence for TEV protease and y denotes the recognition sequence for human rhinovirus 3C protease (HRV3C). Treatment of RGG-x-RGG-y-RGG with a single protease, TEV, removed one RGG domain, resulting in an equimolar mixture of single and tandem RGG. However, treatment with both proteases, TEV and HRV3C, converted the triplet RGG to single RGG (Supplementary Fig. 4A). TEV-treated RGG-x-RGG-y-RGG remained turbid at room temperature, even though its transition temperature was reduced compared to untreated triplet RGG (Fig. 2e and Supplementary Fig. 4B). In contrast, RGG-x-RGG-y-RGG treated with both proteases was soluble at room temperature, only phase separating at temperatures < 15 °C, consistent with the phase behavior of a single RGG domain. RGG-x-RGG-y-RGG functions as a biomolecular AND gate: protein droplets treated with TEV alone remained phase separated at room temperature, whereas further addition of HRV3C protease in a second step caused the droplets to dissolve (Fig. 2f). Logical gating of the REPS system, as demonstrated by this AND gate, could be used to integrate and respond to multiple inputs in a programmable manner.

### Triggered droplet assembly via removal of a solubilizing tag

Full control of droplet phase behavior requires triggerable assembly as well as disassembly. We therefore investigated whether droplet assembly could be mediated by protease activity (Fig. 2g, schematic). We genetically fused maltose-binding protein (MBP) to the N terminus of RGG-RGG, to form MBP-RGG-RGG. MBP has previously been used as a solubility-enhancing tag to prevent phase separation of IDPs such as FUS^9^. The recognition sequence for HRV3C protease was incorporated between MBP and RGG-RGG. In the absence of protease, MBP-RGG-RGG was fully soluble and did not form protein droplets. However, droplets rapidly assembled upon addition of HRV3C protease, which liberated MBP from RGG-RGG (Fig. 2h and Supplementary Fig. 4C). Droplets increased in size, often via fusion of two smaller droplets, and reached their maximum diameter in <2 h (Fig. 2i). A time-dependent turbidity assay at constant temperature (25 °C) confirmed protease-mediated droplet assembly. Treatment of MBP-RGG-RGG with HRV3C protease caused a rapid increase in turbidity, whereas in the absence of protease, no turbidity was detected (Fig. 2j). Therefore, by integrating droplet-forming domains, solubility-enhancing domains, and protease recognition sites, we successfully designed protein compartments capable of triggered assembly and disassembly.

### Permeability of RGG-RGG liquid droplets

**Fig. 3:**
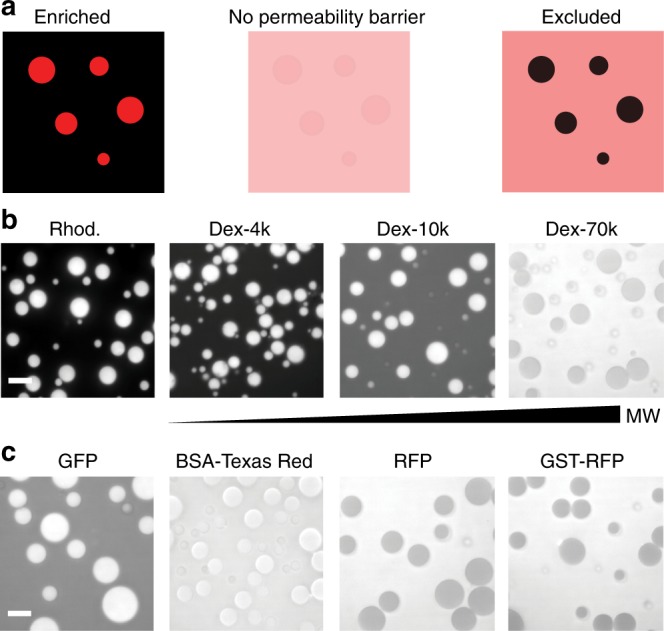
Permeability of RGG-RGG droplets to small molecules and macromolecules. **a** Schematic illustrating permeability of protein droplets to exogenous molecules: a molecule (red) diffusing in solution may become enriched in the droplet phase (left), partition equally between the two phases (middle), or be excluded from the droplets (right). **b** Rhodamine enriches in the droplets, whereas rhodamine-labeled dextrans exhibit size-dependent partitioning—larger molecules permeate the protein droplets less than smaller ones. Dextran 4.4 kDa enriches, while 10 kDa modestly enriches, and 70 kDa is largely excluded. **c** Partitioning of folded proteins into RGG-RGG droplets. None are strongly enriched and some are partially excluded from the RGG-RGG droplet phase. Intensity scale is the same for all micrographs. Scale bars: 10 µm

To harness IDP droplets as synthetic, tunable organelles, it is necessary to understand their inherent permeability to molecules present in the external milieu. Considering their potential uses in cellular engineering, a basic requirement is that the droplets be able to concentrate specific proteins and exclude others (schematic, Fig. 3a). Molecules that are permeable and that have attractive interactions with RGG-RGG will become enriched; solutes that diffuse freely into the droplets but do not interact with RGG-RGG will attain the same concentration inside and outside the organelle; and solutes to which RGG-RGG poses a diffusion barrier will be excluded. One scenario useful for metabolic engineering would be permeability to small molecules, low permeability to endogenous subcellular proteins, and enrichment of targeted cargos. To characterize permeability of RGG-RGG protein droplets, we mixed them with small molecules, dextrans of different molecular weights, and proteins, and then measured solute partitioning using fluorescence microscopy. We found that molecular size inversely correlated with partitioning into RGG-RGG droplets (Fig. 3b). Rhodamine-labeled dextrans of increasing size showed increasing exclusion from the droplets (the enrichment index, or ratio of fluorescence intensity inside to outside the droplets, was approximately 4 for 4.4 kDa dextran and 0.8 for 70 kDa dextran; Supplementary Fig. 5A). Next, we examined the partitioning of proteins whose molecular weights are close to those of typical proteins in a cell. We found that enhanced green fluorescent protein (GFP; approximately 30 kDa) was weakly enriched in the RGG-RGG droplets, approximately 1.6-fold above the continuous phase (Fig. 3c and Supplementary Fig. 5A, B). In contrast, monomeric red fluorescent protein (RFP; 30 kDa), was excluded from the droplets, as was RFP fused to glutathione *S*-transferase (GST-RFP; 55 kDa). Texas Red-labeled bovine serum albumin (BSA; 68 kDa) represented an intermediate case, in which we observed equal fluorescence intensity inside and outside the droplets. These data demonstrate that the unique physicochemical properties of a protein, not just its size, determines its partitioning into RGG-RGG droplets, in agreement with recent reports on other phase-separating IDPs^35^.

### Recruitment of soluble protein cargo to RGG-RGG compartments

**Fig. 4:**
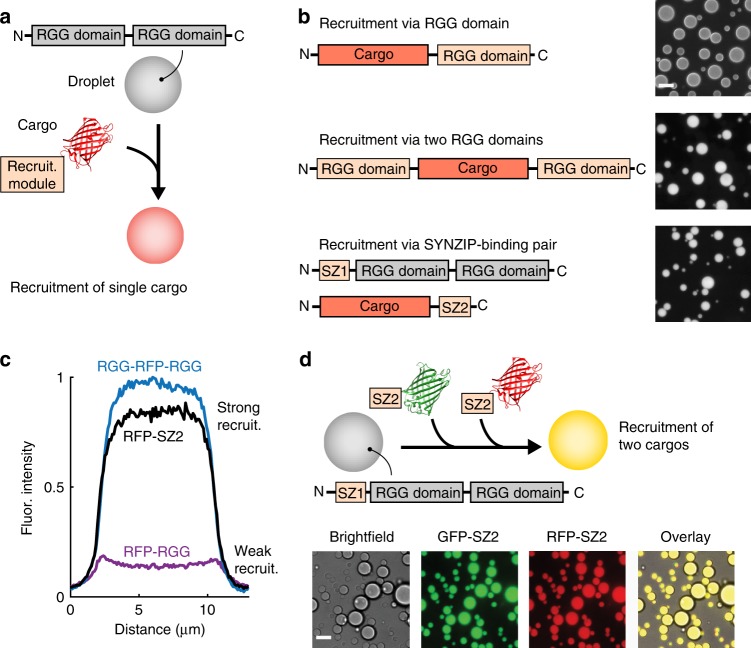
Targeted recruitment and enrichment of cargo proteins in RGG-based compartments. **a** Schematic of cargo recruitment into RGG-RGG liquid droplets. **b** Three strategies for recruitment of model cargo, RFP, that is normally excluded from RGG-RGG droplets: (i) recruitment via a single RGG domain, (ii) recruitment via two RGG domains, and (iii) recruitment via a high-affinity SYNZIP (SZ)-binding pair, in which SZ1 is attached to phase-separating RGG-RGG and its binding partner SZ2 is attached to RFP. Domain schematics and fluorescence micrographs of cargo enrichment are shown. Cargo protein concentration: 1 μM. RGG-RGG or SZ1-RGG-RGG concentration: 6 μM. **c** Quantification of cargo enrichment. Fluorescence intensity line scans of representative droplets show weak recruitment with a single RGG domain, vs. strong and uniform recruitment by two RGG domains or by SZ1/SZ2 binding. **d** Simultaneous recruitment of multiple cargo proteins, GFP-SZ2 and RFP-SZ2, into SZ1-RGG-RGG droplets. Merged image shows co-recruitment (yellow). Scale bars: 10 µm

Next, to control the internal contents of RGG-RGG compartments, we engineered selective recruitment of defined cargos (schematic, Fig. 4a) using three complementary strategies (Fig. 4b). In the first strategy, we attached a single RGG domain to the cargo (cargo-RGG). Second, we attached two RGG domains to the cargo, one at each terminus (RGG-cargo-RGG). Third, to achieve a more modular strategy for recruitment into protein organelles, we tested whether protein interaction motifs, such as the coiled-coil pair SYNZIP1 and SYNZIP2 (SZ1 and SZ2), were sufficient to recruit and concentrate exogenous cargo proteins^36^. We made an N-terminal fusion of SZ1 to RGG-RGG, and a C-terminal fusion of SZ2 to cargo (SZ1-RGG-RGG and cargo-SZ2). As a model cargo, we selected RFP, which is normally excluded from the RGG-RGG droplets.

Upon mixing droplets with 1 µM of the cargo constructs (RFP fused to recruitment tags), fluorescence microscopy (Fig. 4b) revealed that the cargo-RGG only weakly partitioned into RGG-RGG droplets. In contrast, attachment of cargo to two RGG domains, or attachment of SZ1 to the droplet-forming RGG-RGG construct and SZ2 to the cargo, strongly and uniformly recruited RFP (Fig. 4b, c). To quantify this observation, we calculated an enrichment index based on the intensity of fluorescence inside the droplet divided by the intensity outside. For RFP-RGG, we measured a modest enrichment index of approximately 4, whereas RGG-RFP-RGG and RFP-SZ2 had enrichment indices of 27 and 20, respectively (Supplementary Fig. 5C). Importantly, droplets formed by SZ1-RGG-RGG, in addition to recruiting RFP-SZ2, were also capable of simultaneous recruitment of a second protein, GFP-SZ2 (Fig. 4d). This result suggests that multiple distinct proteins, such as a pathway of metabolic enzymes, could potentially be colocalized and concentrated within an individual synthetic organelle.

### Triggered release of cargos from RGG-RGG protein droplets

**Fig. 5:**
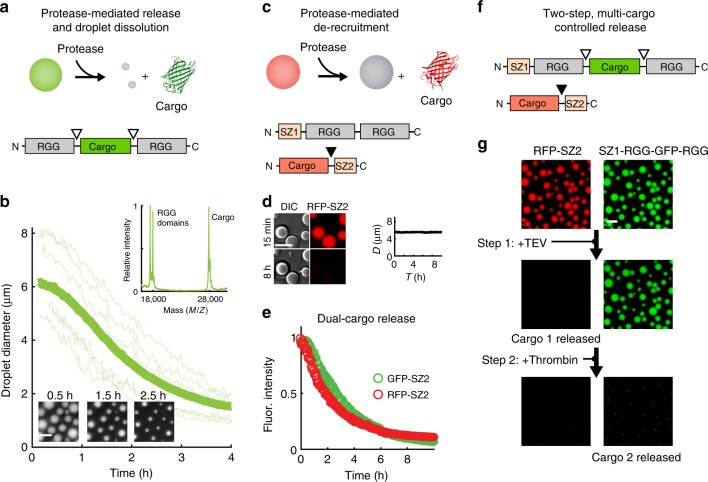
Triggered cargo release from membraneless compartments. **a** Approach 1—protease-mediated release of covalently attached cargo concomitant with droplet dissolution. RGG-cargo-RGG construct; cargo is flanked by thrombin protease cleavage sites. **b** After thrombin treatment (25 nM) of RGG-GFP-RGG, droplets shrank and released the cargo, GFP, into solution. Thick curve: average droplet diameter vs. time for >400 droplets; thin traces: individual droplet shrinking. Inset below: micrographs 0.5, 1.5, and 2.5 h after addition of protease. Insert above: MALDI-TOF shows full-length cargo (GFP), along with the single RGG domains, released upon thrombin treatment. **c** Approach 2—protease-mediated release of tethered cargo; compartments remain intact. Cargo-SZ2 recruited into SZ1-RGG-RGG droplets. Cargo and SZ2 separated by TEV cut site. **d** Near-complete release of RFP cargo 8 h after treatment with TEV protease (0.5 μM) to remove SZ2 recruitment tag. SZ1-RGG-RGG droplets remain intact. **e** Kinetics of dual cargo release from droplets after TEV protease treatment to remove recruitment tag (average of >200 droplets; individual plots shown in Supplementary Fig. 5D). Cargos: GFP-SZ2 and RFP-SZ2 (1 μM each). **f** Two-step, multi-cargo, controlled release. Mixture of cargo2-SZ2 and SZ1-RGG-cargo1-RGG. Cargo 1 (GFP) flanked by thrombin cleavage sites; cargo 2 (RFP) and SZ2 separated by TEV cut site. **g** Initially, compartments contain both cargos, with colocalized green and red fluorescence. Upon treatment with TEV, RFP is released and red fluorescence is lost; protein droplets remain intact and display green fluorescence. Subsequent thrombin treatment liberates the GFP and disassembles the droplets. In all experiments, 5–10 μM RGG-RGG constructs are used. Scale bars: 10 µm

Sequestration and controlled release of proteins has important applications for drug delivery^37,38^ and cellular engineering^39,40^. Therefore, we tested multiple strategies to release protein cargos from RGG-RGG droplets (Fig. 5). First, we released cargo concomitantly with reversing protein phase separation (Fig. 5a). We formed droplets from RGG-cargo-RGG protein that contained thrombin cleavage sites flanking both sides of the cargo, using GFP as a model cargo. Addition of thrombin led to dissolution of the droplets and released GFP by cleaving the RGG-GFP-RGG into fragments of predicted sizes (Fig. 5b). We next tested whether an alternative strategy would allow cargo release while maintaining RGG-RGG droplet integrity (Fig. 5c). We prepared RFP cargo fused to SZ2, with a TEV protease cleavage site between the cargo and recruitment module. This cargo, which is robustly recruited to liquid droplets formed from phase separated SZ1-RGG-RGG protein, was liberated upon treatment with TEV protease (Fig. 5d). Importantly, the SZ1-RGG-RGG droplets remained intact, demonstrating that their phase separation is insensitive to protease-triggered cargo release. Further, we co-recruited two cargos, GFP-SZ2 and RFP-SZ2, to SZ1-RGG-RGG droplets. Both cargos contained TEV cleavage sites between the cargo and recruitment module. Treatment with TEV resulted in simultaneous cargo release (Fig. 5e and Supplementary Fig. 5D). Thus, by fusing a modular recruitment motif to our RGG-RGG platform, and a cognate binding motif to cargo via a protease-cleavable linker, we can program recruitment and release of multiple proteins from synthetic membraneless compartments.

Finally, we combined multiple recruitment and release strategies to enable more complex schemes for controlled release. We prepared a protein termed SZ1-RGG-cargo-RGG, containing two RGG domains, cargo, and a recruitment module (Fig. 5f). We selected GFP as the cargo and incorporated thrombin-cleavable domains flanking the GFP. We mixed SZ1-RGG-cargo-RGG with a second cargo, RFP-SZ2, containing a TEV cut site between the RFP and SZ2. These two proteins assembled to form droplets with colocalization of the two cargos (Fig. 5g). In the first release step, treatment with TEV de-recruited the RFP by liberating it from SZ2. The droplets remained intact and fluoresced green, despite having lost their red fluorescence. In the second release step, thrombin treatment released the GFP cargo while concomitantly dissolving the droplets. This reaction sequence demonstrates multi-step, multi-cargo release strategies built upon the REPS platform. This strategy could be further expanded to controlled release of a third cargo using another SYNZIP pair and a distinct protease cut site.

### Synthetic RGG-RGG organelles in protocells and living cells

**Fig. 6:**
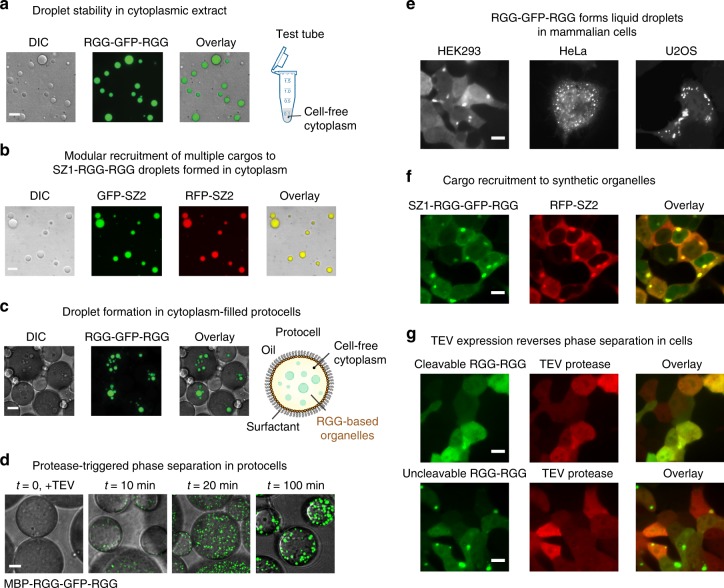
RGG-based synthetic organelles in protocells and living cells.  Scale bars: 10 µm. **a** RGG-GFP-RGG forms stable compartments in model cell cytoplasm prepared from *Xenopus laevis* eggs. **b** Multiple, specific, SZ2-tagged cargos are simultaneously recruited into SZ1-RGG-RGG droplets in the presence of cytoplasm. Undiluted *Xenopus* egg cytoplasmic extract was mixed with SZ1-RGG-RGG (5 µM), GFP-SZ2 (1 µM), and RFP-SZ2 (1 µM). **c** RGG-GFP-RGG protein droplets form in cell-like structures, or protocells. Aqueous phase containing undiluted *Xenopus* egg cytoplasmic extract mixed with RGG-GFP-RGG protein was emulsified within a continuous mineral oil phase containing surfactant. **d** Protease-triggered phase separation in protocells. Aqueous phase containing *Xenopus* egg cytoplasmic extract mixed with 30 µM MBP-RGG-GFP-RGG protein and 0.5 µM TEV protease was encapsulated in emulsions. TEV activity liberates MBP from RGG-GFP-RGG, resulting in triggered formation of RGG-GFP-RGG droplets. **e** RGG-GFP-RGG forms synthetic membraneless organelles following transfection in multiple human cell lines: HEK293, HeLa, and U2OS. **f** Recruitment of exogenous cargo into synthetic organelles via SZ1/SZ2 interaction. RFP-SZ2 (cargo) plasmid was co-transfected with SZ1-RGG-GFP-RGG plasmid in HEK293 cells. **g** TEV expression reverses phase separation and disassembles synthetic organelles in HEK293 cells. RGG-x-GFP-RGG (x = TEV cut site) was co-transfected with RFP-TEV protease. In control experiments using RGG-GFP-RGG that lacks a TEV cut site, droplets form normally and are insensitive to co-expression of RFP-TEV protease

For applications in protocell assembly and metabolic engineering, it is necessary that RGG-RGG droplets assemble and concentrate cargo in the complex cytoplasmic milieu of cells. To test this, we first characterized phase separation and cargo recruitment within a model cell cytoplasm, *Xenopus* egg extract^41^. We found that RGG-cargo-RGG protein formed liquid droplets in this model cytoplasm (Fig. 6a). Additionally, we found that multiple SZ2-tagged cargos could be recruited into SZ1-RGG-RGG organelles (Fig. 6b) in these extracts. Next, we tested whether the organelles would form in protocells—cell-sized compartments filled with cytoplasmic extract. RGG-GFP-RGG formed fluorescent organelles when encapsulated inside these cell-like compartments (Fig. 6c). Additionally, we were able to induce assembly of membraneless organelles by encapsulating MBP-x-RGG-GFP-RGG (x = TEV cut site) immediately after adding TEV protease. TEV protease activity liberated the MBP from RGG-GFP-RGG, resulting in protease-triggered organelle assembly in protocells (Fig. 6d).

To demonstrate the broad utility of our synthetic membraneless organelles, we characterized their properties in living cells. We monitored assembly of protein compartments by transiently transfecting RGG-GFP-RGG in three separate mammalian cell culture lines: HEK293, HeLa, and U2OS. We found micron-sized structures assembled in each cell line (Fig. 6e), demonstrating that our REPS platform phase separates in vivo. Cells were healthy and grew normally, indicating that our biomaterial is orthogonal to cells. The RGG-GFP-RGG organelles localized to the cytoplasm—a useful feature for metabolic engineering—and were distinct from other endogenous organelles (Supplementary Fig. 6A). These organelles are dynamic, recovering rapidly following photobleaching (Supplementary Fig. 6B). Future applications, including metabolic engineering, will require compartmentalization of folded proteins in synthetic organelles in cells. Importantly, SZ1-RGG-GFP-RGG co-transfected with RFP-SZ2 resulted in concentration of soluble RFP cargo in the synthetic organelles (Fig. 6f). This result demonstrates that exogenous protein cargo can be recruited into RGG-based organelles using a small, modular tag. We also reasoned that simply fusing folded protein domains to RGG-RGG could be an alternative approach for colocalizing multiple cargos in synthetic organelles in cells. We therefore co-transfected RGG-GFP-RGG and RGG-RFP-RGG and found that the two fluorescent proteins indeed colocalized in the membraneless organelles (Supplementary Fig. 6C). Finally, we investigated the use of TEV protease to manipulate phase separation in living cells. When we co-transfected TEV protease along with TEV-cleavable RGG-GFP-RGG, the organelles were disassembled and no longer visible (Fig. 6g). In contrast, when we co-transfected TEV protease along with RGG-GFP-RGG that lacked any TEV cut sites, we observed robust formation of synthetic organelles. Therefore, TEV protease can be harnessed as a tool to alter phase separation in cells without affecting cell viability, and in a highly specific manner.

## Discussion

IDPs that self-assemble into liquid droplets have drawn intense interest for their role in subcellular organization and function^1^. Here we utilized an IDP, the RGG domain from LAF-1, to build stimulus-responsive, bio-inspired materials that have applications for engineering synthetic membraneless organelles with novel functionality. We found that engineered multivalent versions of the RGG domain exhibit enhanced phase separation, demonstrated protease-triggered assembly and disassembly of RGG-based droplets, and identified multiple strategies for recruiting cargo proteins to RGG-RGG droplets followed by protease-triggered release of the cargo. RGG-RGG proteins assembled into droplets in vitro in physiological buffer, as well as in cytoplasmic extract. Importantly, using the REPS platform, we formed synthetic organelles in living cells, demonstrated that these organelles can recruit cargo, and showed that their phase separation can be enzymatically reversed. These REPS are modular and genetically encoded for forming dynamic protein microcompartments. Future applications include generating switchable membraneless organelles for metabolic engineering that accelerate biochemical reactions via locally increased concentration of enzymes and substrates. Switchable IDP droplets are also an attractive tool for sequestering and releasing proteins upon stimulation in cells and offer an alternative to inducible systems that rely upon triggerable protein degradation or expression.

Our work provides additional insight into an important physical principle of IDP phase separation: the critical concentration of protein required for liquid–liquid demixing depends strongly on valency. This principle was central to our strategy for engineering controllable phase behavior. Multivalency has been shown to drive the phase separation of folded protein domains, such as SH3 and its ligand PRM, and higher valencies lowered the critical concentration for droplet formation^30^. However, it was unclear whether this principle also extended to phase separation of conformationally heterogeneous IDPs with weak, non-stereospecific interactions. Our results indicate that the increased avidity between molecules of tandem or triplet RGG indeed give rise to robust liquid–liquid demixing at lower protein concentration. Our data are consistent with recent studies on synthetic protein polymers with UCST phase behavior, in which the transition temperature increased as the number of repeat units in the polymer increased^23^.

The synthetic RGG-RGG protein offers several advantages as a platform for engineering programmable membraneless organelles. In contrast to full-length LAF-1, which contains a helicase domain, RGG-RGG is a minimal scaffold that avoids unwanted pleiotropic effects associated with expression of the full-length protein. RGG-RGG is much smaller than LAF-1 and contains no cysteines, which facilitates recombinant expression and bioconjugation. Other recent reports elucidated mechanisms that control biological phase separation^24^, droplet composition^26^, and intracellular formation of solid hydrogels^42^, but our work is unique in demonstrating a minimal, genetically encoded, robust platform for inducible assembly, disassembly, and targeted cargo recruitment into liquid-phase synthetic membraneless organelles suitable for cellular engineering. RGG-RGG does not require RNA to phase separate or to maintain a dynamic, liquid state. Only a single protein is required for phase separation, in contrast to systems that require two interacting species to phase separate^26,30^. Likewise, RGG-RGG-RGG is a promising platform for engineering synthetic organelles and offers additional programmability.

IDP phase behavior is sensitive to temperature and salt concentration; however, in mammalian cells, it is not feasible to trigger droplet assembly or disassembly by drastically altering salt concentration or temperature, which would be toxic to the cell. Enzyme-triggered self-assembly of synthetic materials, such as hydrogels and nanofibers, has been successfully implemented in vitro and in vivo^43–46^. We therefore developed a biocompatible toolkit for programmable IDP phase separation based on enzyme activity. Using protease activity to control RGG-RGG valency or the presence of a solubility-enhancing tag, we developed a minimal system whose phase behavior can be reversed. This feature affords temporal control of droplet phase behavior, which is desirable if a synthetic membraneless organelle is toxic or should be present during only one phase of the cell cycle. Additionally, logical gating of the REPS system could be employed to integrate and respond to multiple signals inside a cell. We also developed multiple protease-based strategies to trigger cargo release from these membraneless organelles, using TEV or thrombin. As we and others have demonstrated, proteases such as TEV can be expressed in cells without interfering with cell function^33^. Recent excellent work demonstrated that IDP phase behavior can also be controlled optogenetically^27^. However, in that approach, phase separation required continuous blue light illumination, whereas in our REPS system, a single stimulus triggers assembly and phase separation, and a second stimulus mediates disassembly and mixing.

The ability to recruit soluble proteins into RGG-RGG droplets provides a paradigm for future applications in metabolic engineering. Researchers have typically used two approaches to concentrate sets of enzymes in a metabolic pathway. The first is to recruit multiple enzymes to a scaffold, which is present throughout a cell^16,22^, and the second is localization to endogenous organelles, such as peroxisomes or mitochondria^12–14^. Synthetic membraneless organelles expand the toolkit for locally concentrating proteins, and our results demonstrate the feasibility of simultaneous recruitment of multiple proteins. RGG-RGG droplets are permeable to small molecules, have low permeability to non-recruited proteins, and can be engineered to selectively recruit proteins of interest. Our results therefore show that the RGG-RGG IDP platform has many characteristics necessary for applying minimal synthetic membraneless organelles in metabolic engineering.

Our results open a number of paths for future investigations aimed at expanding the capacity of this controllable phase separation platform. First, our work and other recent research^47^ hint at strategies for altering droplet density, permeability, and viscosity. These biophysical parameters will likely affect the performance of synthetic organelles in metabolic engineering applications. For instance, engineered permeability may further enhance compartment specificity, and viscosity may influence reaction rates. Second, in addition to recruiting multiple enzymes to the same droplet, enzyme activity could be controlled reversibly through the assembly or disassembly of split enzymes^33,48^. Split enzymes could also be harnessed as a strategy for improving spatial and temporal control in our REPS system. Assembly or disassembly of RGG-based droplets could be triggered by split TEV, whose activity is reconstituted only upon adding a photo-caged dimerizer and exposing it to light^49^.

In conclusion, we have demonstrated how sequence-encoded phase behavior of droplet-forming IDPs can be harnessed as bio-inspired materials, pointing toward their use as synthetic membraneless organelles in cell and protocell engineering. Our expanded understanding of the self-assembly behavior and soft matter properties of phase-separating IDPs provides paradigms for future applications that build upon our results.

## Methods

### Cloning

The gene encoding the RGG domain was amplified by PCR from *LAF-1*. The full-length *LAF-1* gene was a gift of Shana Elbaum-Garfinkle and Clifford Brangwynne, Princeton University^2^. SYNZIPs 1 and 2 (SZ1 and SZ2) were ordered as synthetic gene blocks from IDT based on their published sequences^36^. Plasmids were constructed using standard restriction enzyme cloning techniques or In-Fusion cloning (Takara Bio). Genes were cloned into a pET vector in frame with either a C-terminal 6-His tag (for constructs containing the RGG domain) or N-terminal 6-His tag (for the cargo-SZ2 constructs). In protease-cleavable RGG-RGG constructs, protease cut sites were inserted between the RGG domains. Linkers were not required, as RGG domains are intrinsically disordered and protease activity was high even in the absence of linkers. Sequences were verified by Sanger sequencing (GENEWIZ or University of Pennsylvania DNA Sequencing Facility).

### Protein purification for RGG-based constructs

Recombinant plasmids were transformed into BL21(DE3) *Escherichia coli* strain for protein expression. Cultures were grown in Terrific Broth media at 37 °C until reaching OD_600_ = 0.5–0.8 and were then induced with 0.5 mM isopropyl β-d-1-thiogalactopyranoside (IPTG), followed by overnight expression at 18 °C while shaking at 225 rpm. Bacterial pellets were resuspended in buffer (listed below) and lysed by sonication. The lysate was clarified by centrifugation at 15 000 × *g* for 30 min. To prevent phase separation, RGG-RGG lysates were centrifuged at 40 °C. Proteins were purified on an AKTA FPLC at room temperature using 1 mL Ni-NTA His-Trap columns (GE Healthcare Life Sciences). For RGG-RGG constructs, lysis buffer was 1 M NaCl, 20 mM Tris, 20 mM imidazole, protease inhibitor (EDTA-free; Roche), pH 7.5 and used at sufficient volume such that RGG-RGG remained below its critical concentration for phase separation. The compositions of the remaining buffers were as follows: Lysis buffer for single RGG constructs: 500 mM NaCl, 20 mM Tris, 20 mM imidazole, protease inhibitor, pH 7.5. Wash buffer: 500 mM NaCl, 20 mM Tris, 20 mM imidazole, pH 7.5. Elution buffer: 500 mM NaCl, 20 mM Tris, 500 mM imidazole, pH 7.5.

RGG-RGG-RGG phase separates even under conditions of elevated temperature and high NaCl concentration, so it was purified as follows: bacterial pellets were resuspended in lysis buffer (150 mM NaCl, 20 mM Tris, protease inhibitor, pH 7.5), then lysed by sonication and centrifuged at 15 000 × *g* for 30 min at 4 °C. RGG-RGG-RGG phase separated under these conditions and was predominately found in the pellet. The supernatant was discarded, and the pellet was resuspended in urea buffer (8 M urea, 1 M NaCl, 20 mM Tris, and 20 mM imidazole, pH 7.5). After a second round of centrifugation (at 30 °C), the supernatant was retained and applied to Ni-NTA resin that had been equilibrated with urea buffer. After binding, the column was first washed with urea buffer, then washed with urea-free buffer (1 M NaCl, 20 mM Tris and 20 mM imidazole, pH 7.5). Purified protein was eluted with 1 M NaCl and 500 mM imidazole, pH 7.5.

Proteins were dialyzed overnight into high-salt buffer (500 mM NaCl and 20 mM Tris, pH 7.5) using 7 or 10 kDa molecular weight cut-off membranes (Slide-A-Lyzer, Thermo Fisher). RGG-RGG and RGG-RGG-RGG were dialyzed at elevated temperature (42 and 55 °C, respectively) to inhibit phase separation, because phase-separated proteins sedimented and bound to the dialysis cassette. Single-use protein aliquots were flash-frozen in liquid nitrogen and stored at −80 °C.

Protein concentrations were determined by measuring absorbance at 280 nm (Nanodrop; Thermo Fisher). Proteins were mixed 1:1 in 8 M urea to inhibit phase separation during concentration measurements.

### Protein purification for non-IDP proteins

Recombinant plasmids for His6-GFP, His6-RFP, GST-RFP, and His6-TEV were transformed into Rosetta BL21(DE3) pLysS *E. coli* strain for protein expression. One-liter cultures of lysogeny broth containing ampicillin were grown at 37 °C until reaching OD_600_ = 0.3, then transferred to 16 °C and grown for 30 min until OD_600_ = 0.55–0.65, and finally induced with 0.5 mM IPTG. Cultures were grown overnight at 16 °C. Bacterial pellets were resuspended in standard lysis buffer containing 150 mM NaCl, 50 mM Tris (pH 8), and 5% glycerol. Prior to sonication, bacterial cells suspended in lysis buffer were supplemented with Complete EDTA protease inhibitors and 5 mM 2-Mercaptoethanol (Roche). Samples were further supplemented with 0.1% CHAPS, 2 mM MgCl_2_, and DNAse to aid breakdown of bacterial cell wall and genomic DNA. Cells were lysed by three rounds of freeze-thaw and sonication using a Branson Sonifier. Lysates were clarified by centrifugation at 20 000 × *g* for 20 min.

For His-tagged proteins, clarified supernatant was incubated with 1 mL Ni-NTA-Superflow beads (Qiagen), placed in a disposable column, and washed with 25 mL each of standard buffer, high-salt buffer, and 15 mM imidazole wash buffer. Pure protein was eluted at a concentration range of 3–5 mg/mL in standard buffer containing 250 mM imidazole, then dialyzed overnight into a final buffer consisting of 150 mM NaCl, 25 mM Tris (pH 8), 10% glycerol, and 1 mM dithiothreitol (DTT). Dialyzed proteins were supplemented with 1 mM TCEP, aliquoted, flash-frozen in liquid nitrogen, and stored at −80 °C.

For GST-tagged proteins, clarified supernatant was incubated with 2 mL of glutathione agarose (Pierce) and washed repeatedly using a standard buffer. Pure protein was eluted in a standard buffer containing 15 mM reduced glutathione, then dialyzed overnight into a final buffer containing 150 mM NaCl, 25 mM Tris, pH 8, 10% glycerol, and 1 mM DTT. Dialyzed proteins were supplemented 1 mM TCEP, aliquoted, flash-frozen in liquid nitrogen, and stored at −80 °C.

Protein concentrations were determined using Bradford Plus protein reagent. All purification steps with non-IDP proteins were carried out on ice or at 4 °C. Purified proteins were frozen at stock concentrations of 50–200 μM.

### Sample preparation for turbidity and microscopy experiments

RGG-RGG protein aliquots were thawed above the UCST to ensure the protein was soluble. Phase separation was induced by mixing RGG-RGG (stored in 500 mM NaCl and 20 mM Tris, pH 7.5) with no-salt buffer (0 mM NaCl and 20 mM Tris, pH 7.5) to obtain a solution containing 150 mM NaCl and 20 mM Tris, pH 7.5. Protein concentrations were adjusted as necessary by diluting further with same buffer conditions. Thrombin digestion experiments were conducted using restriction-grade thrombin (Novagen/EMD Millipore). TEV experiments were conducted using either ProTEV Plus (Promega) protease or with TEV protease purified in our lab from the pRK793 plasmid^50^. Both TEV proteases had similar activity. HRV3C protease experiments were conducted using either HRV3C protease purchased from Thermo Fisher, which contained a GST tag (used for the triplet RGG experiments), or using HRV3C protease purchased from Takara Bio, which was not GST-tagged (used for MBP-RGG-RGG experiments). Rhodamine B and rhodamine-labeled dextrans (4.4, 10, and 70 kDa) were purchased from Sigma-Aldrich. BSA-Texas Red was purchased from TherrmoFisher. In their respective experiments, protease, dextrans, or fluorescent proteins were mixed with RGG-RGG protein (6 μM) at the same time as inducing phase separation. Final concentrations were approximately 0.01 mg/mL for rhodamine B and 10 mg/mL for rhodamine-labeled dextrans. 6His-GFP and 6His-RFP were tested at 1 and 10 μM, and GST-RFP and BSA-Texas Red at 10 μM.

### Turbidity assays

Turbidity assays were performed on a UV-Vis spectrophotometer (Cary 100 Bio; Varian) equipped with a multicell Peltier temperature controller. Samples (in quartz cuvettes with 1 cm path length; Thorlabs) were first equilibrated above the transition temperature, and the instrument was blanked. Samples were then cooled at a rate of 1 °C/min until reaching 4 °C. Throughout the experiment, absorbance was measured at *λ* = 600 nm every 0.5 °C. The samples changed from clear solutions to turbid suspensions upon cooling below the UCST.

### MALDI-TOF mass spectrometry

The molecular weights of protein constructs and digested fragments were confirmed using MALDI-TOF mass spectrometry. Samples were spotted on an MTP 384 polished steel target plate (Bruker). Spots consisted of 1 μL of protein solution and 1 μL matrix solution (10 mg/mL sinapinic acid dissolved in 50:50 acetonitrile:water with 0.1% trifluoroacetic acid). Spectra were collected on an Ultraflextreme MALDI-TOF mass spectrometer (Bruker).

### Tissue culture and transfection

HEK293, HeLa, and U2OS cells were plated at a density of 75 000 cells/well in 0.5 mL Dulbecco’s modified Eagle’s medium (supplemented with 10% fetal bovine serum, glutamine, and pen/strep) in a 24-well glass-bottom plate (#1.5 cover glass; Cellvis) and incubated using standard tissue culture methods at 37 °C with 5% CO_2_ and saturating humidity. After 24 h, the cells were transfected with the plasmids indicated below using the X-tremeGENE 9 DNA transfection reagent (Roche) according to the manufacturer’s instructions. Briefly, each transfection reaction was set up by combining 200 µL Opti-Mem (Gibco), plasmid DNA, and the transfection reagent (at a concentration of 1 µL/µg DNA) and gently mixing in a 1.5 mL tube. The transfection mixture was incubated at room temperature for 20 min. Following incubation, the transfection mixture was added dropwise into each well and incubated under standard tissue culture conditions for 24–48 h before imaging.

Plasmids for transfection were constructed by inserting genes for RGG-GFP-RGG, RGG-RFP-RGG, SZ1-RGG-GFP-RGG, RFP-SZ2, or RFP-TEV into the pcDNA3.1 backbone. Plasmids encoding RGG-GFP-RGG (with and without TEV cleavage site), SZ1-RGG-GFP-RGG, and RGG-RFP-RGG were transfected using 500 ng plasmid/well. Plasmids encoding RFP-SZ2 and RFP-TEV were transfected using 25 ng plasmid/well.

For live-cell staining experiments, Hoechst 33342 (BD Pharmingen) was used to visualize nuclei and LysoTracker Red DND-99 (Thermo Fisher) was used to visualize lysosomes. Staining was performed according to the manufacturers’ instructions.

### Microscopy

Images were collected on a Leica DMi8 inverted microscope equipped with a confocal spinning disk unit (Spectral Applied Research) using a ×63, 1.4 numerical aperture (NA) plan-apochromatic oil-immersion objective and sCMOS camera (Orca Flash 4.0; Hamamtsu). For fluorescence imaging, GFP constructs were illuminated with a 488 nm laser and red fluorescent constructs were illuminated with a 561 nm laser. For transmitted light imaging, a 0.55 NA condenser was employed, and samples were illuminated with an LED (ScopeLED).

Protein samples were placed in 16-well glass-bottom dishes (#1.5 glass thickness; Grace Bio-Labs) that were passivated to minimize wetting of protein droplets to the surface. For protease-mediated droplet dissolution and cargo release experiments, the glass surface was passivated by overnight incubation with 1 mg/mL PLL–PEG (20 kDa–2 kDa; SuSoS or custom synthesized). For all other imaging experiments, the glass was passivated by overnight incubation with 5% Pluronic F-127 (Sigma-Aldrich). Coated chambers were thoroughly rinsed with buffer prior to the addition of protein solutions.

### Droplet image analysis

Image segmentation and analysis was performed using custom-written code in MATLAB. For each frame of the videos, the circular Hough transform identified droplets and determined their size and position. Fluorescence intensity within a droplet was calculated by summing the values of pixels circumscribed by the droplet. Individual droplets were followed over time using a particle tracking routine^51,52^. The accuracy of the circular Hough transform becomes limited when droplet size ≤ 10 pixels (approximately 1 μm for the imaging conditions used here).

### Fluorescence recovery after photobleaching

FRAP experiments were conducted on an Olympus inverted microscope equipped with a Yokogawa spinning disk confocal unit, an iLas2 targeted laser system for photobleaching (Roper Scientific), and an iXon3 EMCCD camera (Andor). Bleaching was performed with a 405 nm laser and imaging with a 488 nm laser, using a ×60/1.2 NA water-immersion objective or ×100/1.4 NA oil-immersion objective. FRAP experiments and analysis on RGG-RGG were conducted similarly to published studies on LAF-1^2^ to facilitate comparison. For FRAP experiments on purified proteins in vitro, circular regions with radius *r* between 1.5 and 2 μm were bleached at the center of protein droplets whose diameters were approximately 10 times the bleach radius. For FRAP experiments in living HEK293 cells, the entire droplets were bleached. Recovery curves were corrected for photobleaching, normalized, and then fit to *f*(*t*) = *A*(1 − e^−*t/τ*^), where *τ* is the recovery timescale. The diffusion coefficient was approximated as *D* ≈ *r*^2^/*τ*.

### *Xenopus* egg cytoplasmic extracts and protocell structures

Cytostatic factor-arrested cytoplasmic extracts were prepared from freshly laid eggs of *Xenopus laevis*, as described^41,53–55^. Briefly, eggs in metaphase of meiosis II were collected, dejellied, and fractionated by centrifugation. The cytoplasmic layer was isolated, supplemented with 10 μg/mL of protease inhibitors (leupeptin, pepstatin, and chymostatin), 20 μM cytochlasin D, and a creatine phosphate and ATP energy regeneration mix, and stored on ice for up to 6 h. This cytoplasmic extract contains the proteins, organelles, and subcellular structures present in an intact cell and is competent to carry out in vitro many biochemical processes related to the cell cycle.

Protocell-like structures were formed by emulsifying cytoplasmic extract into a mixture of oil and surfactant. The aqueous dispersed phase consisted of *Xenopus* egg cytoplasmic extract supplemented with RGG-RGG proteins (resulting in <30% dilution of the *Xenopus* extract). The continuous oil phase consisted of mineral oil containing 50 mg/mL Cithrol DPHS (Croda) added as the emulsifier. Water-in-oil emulsions were formed by dispensing 2 μL of cytoplasmic extract mixture into 200 μL of the oil phase, followed by pipetting to break the aqueous phase into cell-size droplets.

To analyze phase separation and formation of membraneless organelles in cytoplasmic extracts and protocells (Fig. 6a–d), the following conditions were used: For Fig. 6a: undiluted *Xenopus* egg cytoplasmic extract was mixed with protein to generate a final concentration of 25 µM RGG-GFP-RGG. For Fig. 6b: undiluted *Xenopus* egg cytoplasmic extract was mixed with protein to generate final concentrations of 5 µM SZ1-RGG-RGG and 1 µM each of GFP-SZ2 and RFP-SZ2. For Fig. 6c: undiluted *Xenopus* egg cytoplasmic extract was mixed with 25 µM RGG-GFP-RGG protein and emulsified within a continuous mineral oil phase containing Cithrol DPHS surfactant. In Fig. 6a–c, samples were imaged at 1 h after assembly. For Fig. 6d: aqueous phase containing *Xenopus* egg cytoplasmic extract mixed with 30 µM MBP-RGG-GFP-RGG protein and 0.5 µM TEV protease was emulsified as described above. Samples were imaged over 100 min.

### Data availability

The sequence data (in GenBank format) for the plasmids used in this study are available in Supplementary Data 1. Other data supporting the findings in the manuscript and custom code used for data analysis are available from the corresponding authors upon reasonable request.

## Electronic supplementary material

Supplementary Information

Description of Additional Supplementary Files

Supplementary Data 1
