# Supplementary material for: Controllable protein phase separation and modular recruitment to form responsive membraneless organelles
Source: Nat Commun. 2018 Jul 30;9:2985. doi: 10.1038/s41467-018-05403-1 (PMC6065366; doi:10.1038/s41467-018-05403-1)
Supplement: Supplementary file 1 — Supplementary Information [file 41467_2018_5403_MOESM1_ESM.pdf]

Controllable Protein Phase Separation and Modular Recruitment to Form Responsive Membraneless Organelles

Schuster et al.

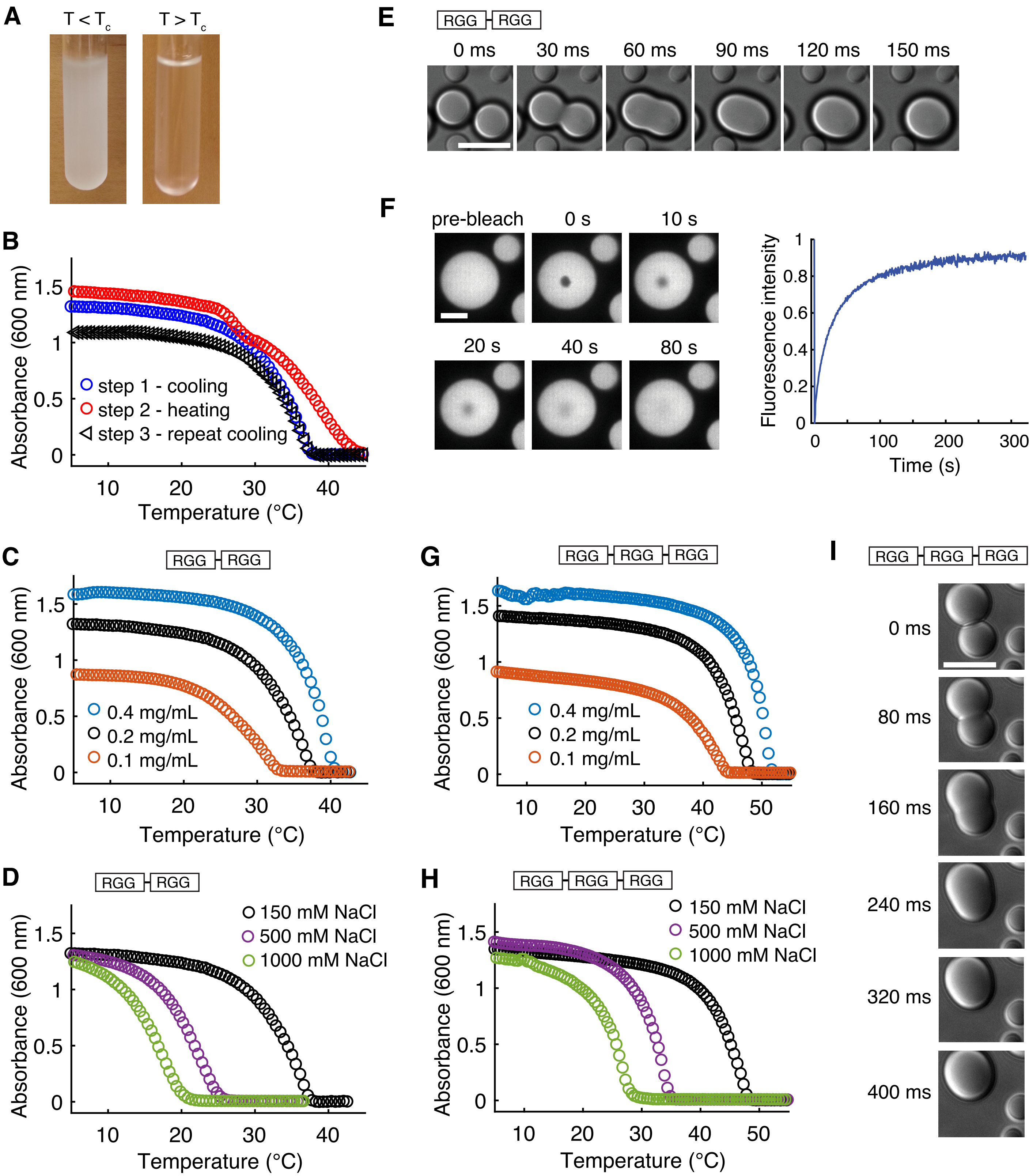

Supplementary Figure 1. Material properties of RGG-RGG and RGG-RGG-RGG. (A-B) RGG-based proteins form droplets that can be cycled with temperature. (A) Photographs of RGG-RGG solutions at temperatures less than (left side: turbid, two phase) and greater than (right side: clear, single phase) the critical temperature. (B) RGG-RGG phase separation can be cycled, as monitored by temperature-dependent turbidity assays. Higher absorbance corresponds to an increase in turbidity upon formation of protein droplets. Turbidity assay shows RGG-RGG droplet assembly upon cooling (blue), disassembly upon heating (red), and subsequent droplet reassembly upon recooling (black). RGG-RGG concentration was approximately 0.2 mg/mL (6 μM) in buffer containing 150 mM NaCl, pH 7.5.

(C-D) Dependence of RGG-RGG phase behavior on salt and protein concentrations. (C) Transition temperature for phase separation increases with RGG-RGG protein concentration. Protein tested at approximately 0.1 to 0.4 mg/mL (3 to 12 μM), in buffer containing 150 mM NaCl, pH 7.5. (D) Transition temperature of RGG-RGG decreases with increasing NaCl concentration. RGG-RGG present at 0.2 mg/mL (6 μM). **(E-F) RGG-RGG protein droplets exhibit dynamic, liquid-like properties.** (E) RGG-RGG droplets rapidly fuse and return to spherical shape within approximately 100 ms. Scale bar: 10 μm. (F) Fluorescence recovery after photobleaching of a small circular region within an RGG-RGG droplet (containing 5% RGG-GFP-RGG for imaging). The half-time for recovery is approximately 20 s, and greater than 90% of the fluorescence recovers after 5 min. Scale bar: 10 μm. **(G-I) Characterization of RGG-RGG-RGG.** (G) Transition temperature increases with RGG-RGG-RGG protein concentration. Protein tested at approximately 0.1 to 0.4 mg/mL (2 to 8 μM), in buffer containing 150 mM NaCl, pH 7.5. (H) Transition temperature of RGG-RGG-RGG decreases with increasing NaCl concentration. RGG-RGG-RGG present at 0.2 mg/mL (4 μM). (I) RGG-RGG-RGG droplets exhibit dynamic liquid properties, fusing and returning to spherical shape within $<$ 400 ms. Scale bar: 10 μm.

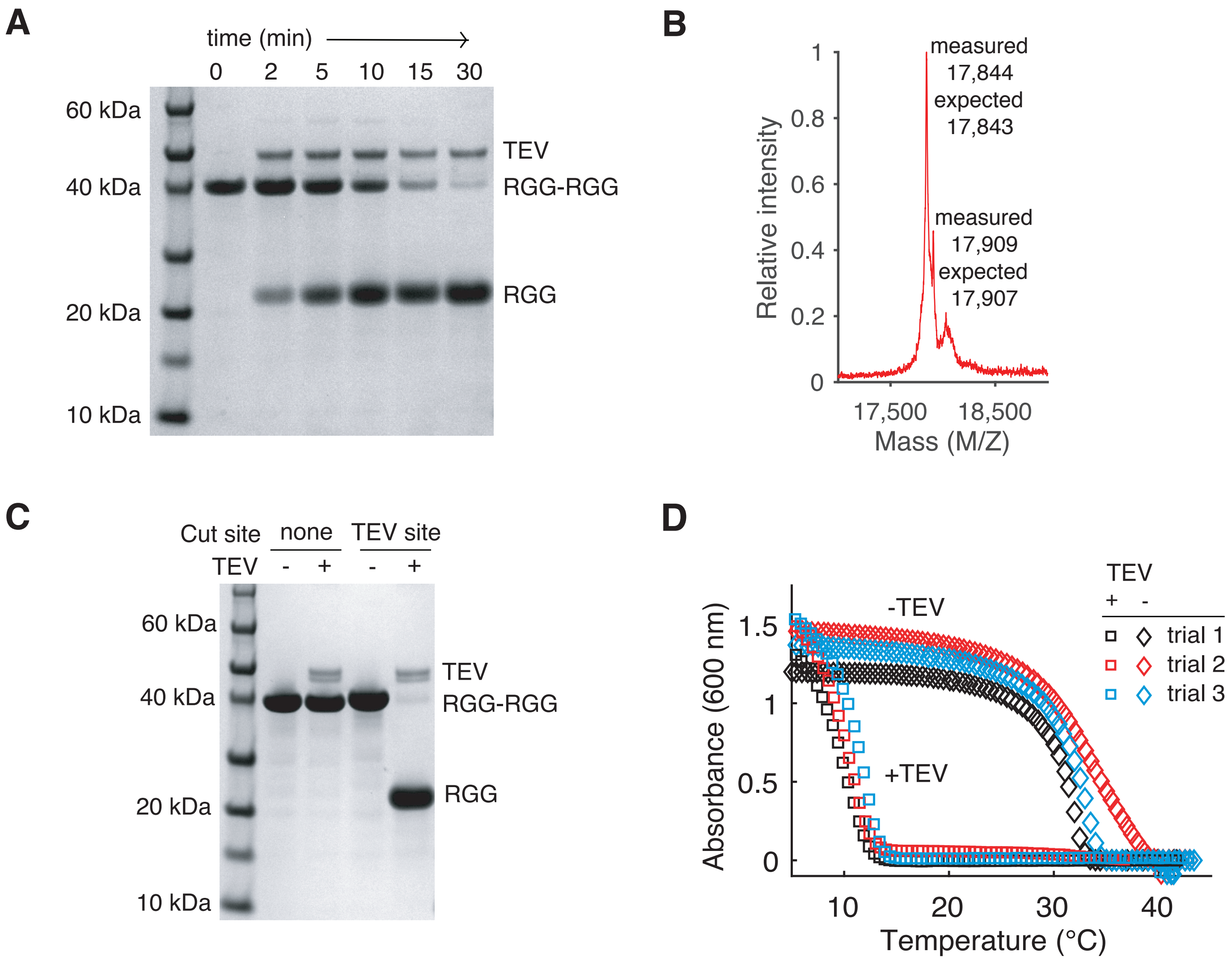

Supplementary Figure 2. TEV protease-dependent cleavage and disassembly of RGG-x-RGG protein droplets (x = ENLYFQG, the recognition and cleavage site for TEV). (A) Timecourse of RGG-x-RGG (6 µM) cleavage with TEV protease (1 µM) shown on Coomassie-stained SDS-PAGE gel. (B) MALDI-TOF mass spectrum of TEV-treated RGG-x-RGG confirms the expected molecular weights of digestion products. (C) TEV is a highly sequence-specific protease. TEV treatment of RGG-RGG lacking a cut site results in no digestion. In contrast, RGG-x-RGG treatment with TEV results in digestion of approximately 100% of RGG-x-RGG. RGG tandem concentration was 6 µM, TEV concentration was 1 µM, and the digests proceeded for 2 h. (D) The phase transition temperature of RGG-x-RGG (6 µM) treated with TEV (1 µM) is markedly reduced compared to that of untreated RGG-x-RGG. Three independent experiments are shown. The digested protein exhibits similar phase behavior to single RGG (Fig. 1C).

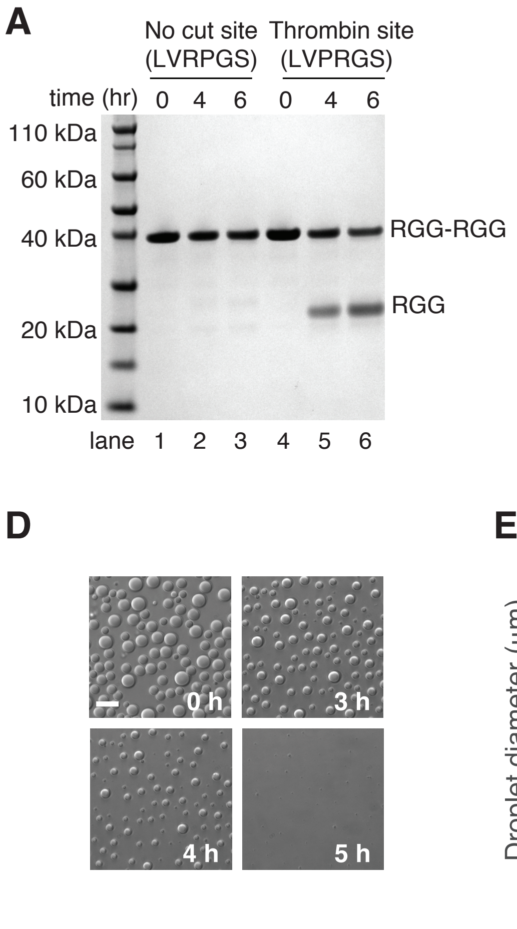

Supplementary Figure 3. Thrombin protease-dependent cleavage and disassembly of tandem RGG droplets. In place of a TEV site, tandem RGG was engineered with the canonical thrombin cleavage and recognition sequence (z = LVPRGS) to generate RGG-z-RGG. To serve as a negative control, a construct was generated with the thrombin cut site mutated by transposing the P and R to produce RGG-z'-RGG, where z' = LVRPGS. (A) Cleavage of tandem RGG depends on presence of protease cut site. Coomassie-stained SDS-PAGE gel showing timecourse of digestion following the addition of thrombin (4 nM) to RGG-z-RGG (3 μM) or negative control RGG-z'-RGG (3 μM). After 6 h of treatment with thrombin, substrate containing LVPRGS cut site is approximately 50% cleaved, whereas the LVRPGS negative control exhibits minimal digestion (based on MALDI-TOF, we attribute the faint bands in lane 3 to thrombin cleavage between R155 and S156 in the RGG domain). (B) MALDI-TOF spectrum of cleavage products from thrombin-treated RGG-z-RGG shows expected masses. (C) The phase transition temperature of thrombin-treated RGG-z-RGG (approximately 80% digested) is markedly reduced compared to that of untreated RGG-z-RGG. (D) Thrombin-triggered droplet dissolution monitored by time-lapse microscopy. Images of the droplets at timepoints 0, 3, 4, and 5 h after adding thrombin. Scale bar: 10 μm. (E) Analysis of the droplet dissolution from time-lapse videos. Comparison of droplet dissolution for RGG-z-RGG vs. control lacking a cut site (RGG-z'-RGG), both treated with thrombin. Diameter vs. time for RGG-z-RGG droplets shown as representative individual traces (thin red lines) and population average (thick red line), compared to population average for RGG-z'-RGG droplets (thick gray line). Both population averages are of > 500 droplets. Thrombin cleavage reverses phase separation of RGG-z-RGG. Droplets formed by RGG-z'-RGG remain phase separated for > 5 hours. (F) Traces show fractional volume released from thrombin-treated RGG-z-RGG droplets plotted against time for 40 individual droplets, as well as population average (thick red line). The curves suggest approximately linear kinetics for dissolution.

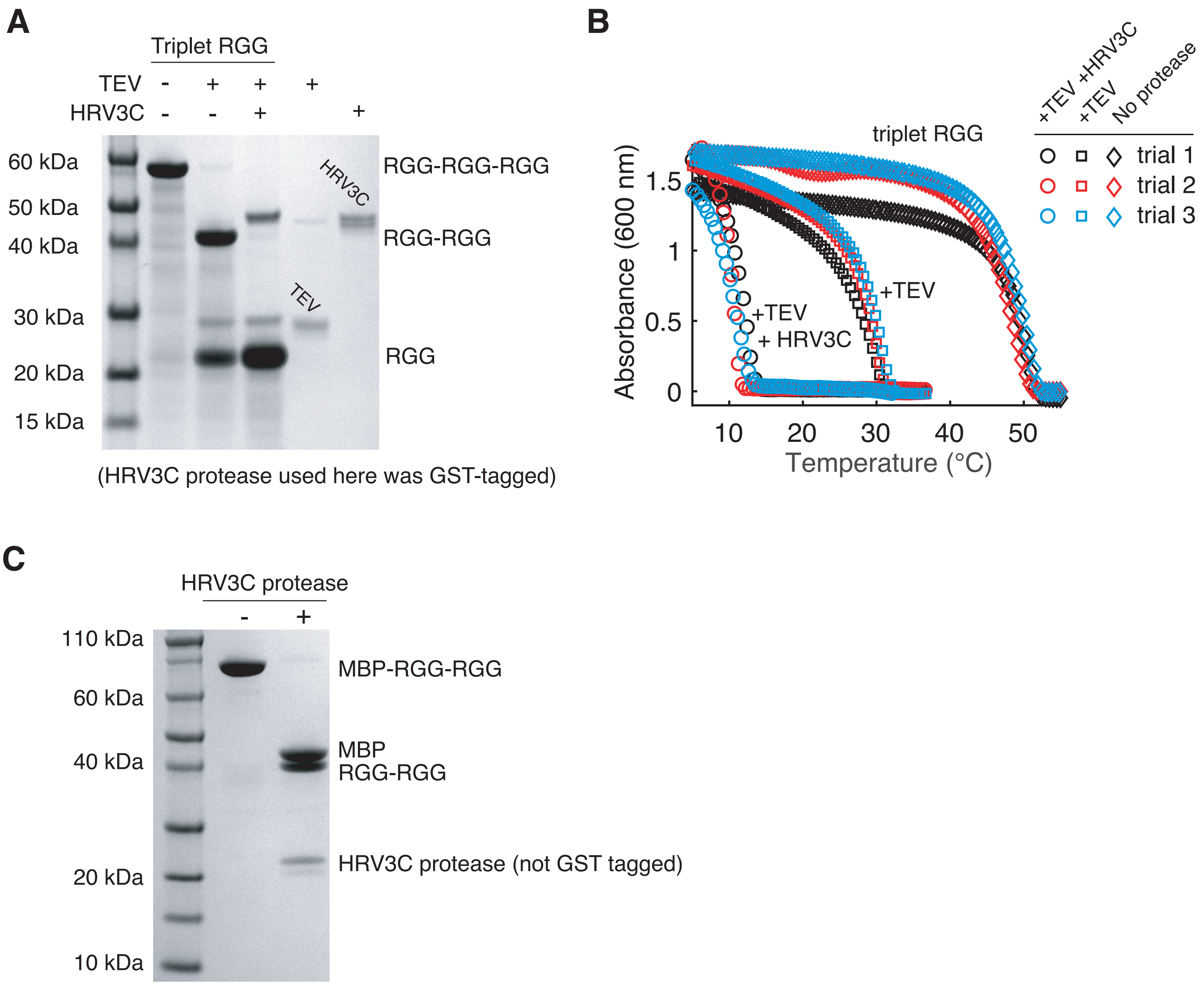

Supplementary Figure 4. Effect of protease treatment on triplet RGG and MBP-RGG-RGG. (A) Effect of protease treatment on RGG-x-RGG-y-RGG (6 µM; x = TEV cut site; y = HRV3C cut site = Leu-Glu-Val-Leu-Phe-Gln-Gly-Pro) shown on a Coomassie-stained SDS-PAGE gel. Treatment with TEV removes only the first RGG domain, resulting in an equimolar mixture of single and tandem RGG, whereas treatment with both TEV and HRV3C (approximately 0.5 µM each) converts the triplet RGG to single RGG. (B) Protease treatment alters phase behavior of RGG-x-RGG-y-RGG (6 µM). Treatment with TEV reduces the phase transition temperature, yet protein remains phase separated at room temperature. Treatment with both TEV and HRV3C results in phase separation only at temperatures < 15 °C. Three independent experiments are shown. (C) Protease removes maltose-binding protein (MBP) tag from MBP-RGG-RGG. Coomassie-stained gel showing cleavage of 5 µM MBP-y-RGG-RGG (y = HRV3C site) with HRV3C protease. Gel shows that protease treatment liberates approximately 100% of MBP from RGG-RGG.

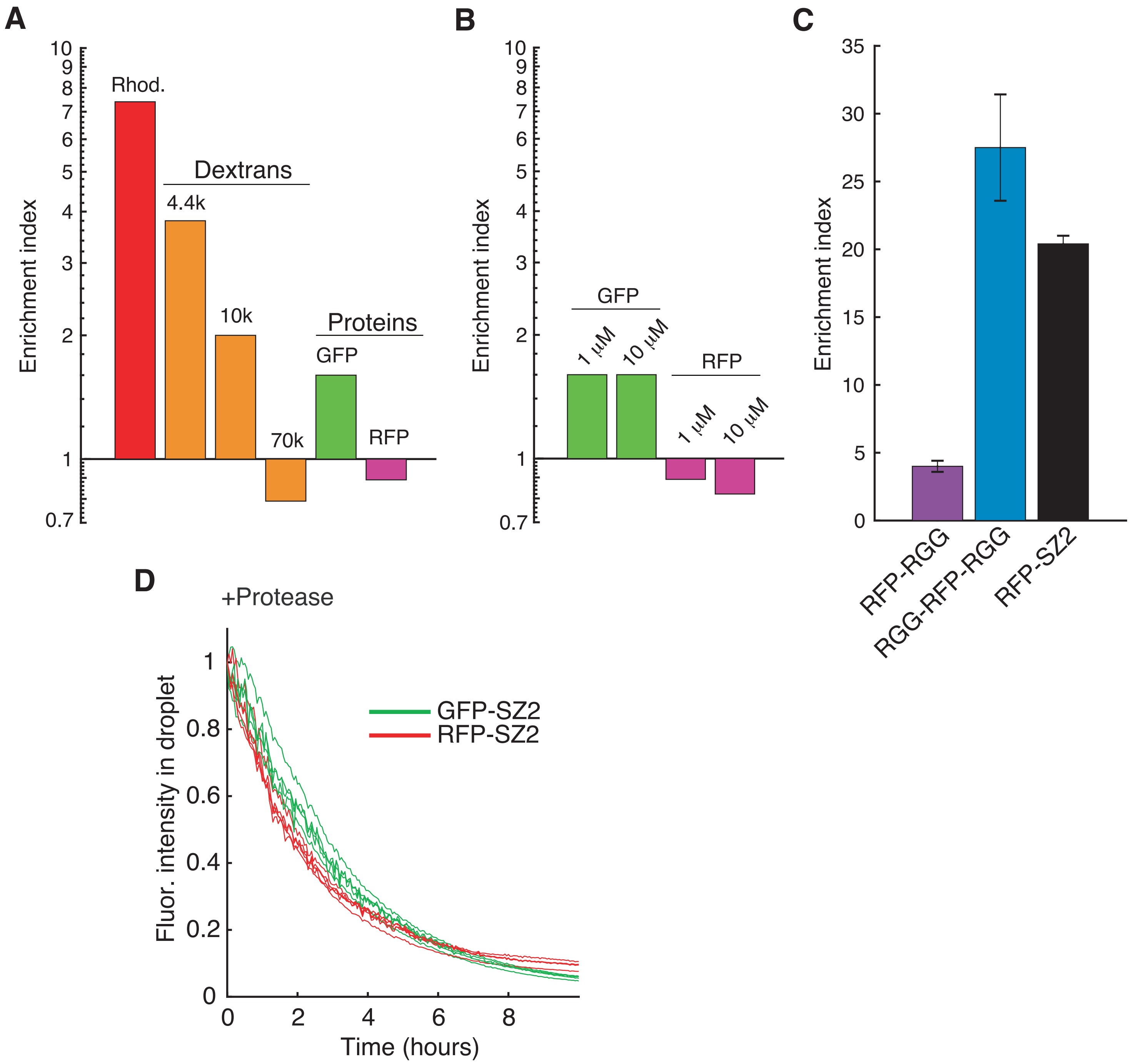

Supplementary Figure 5. (A-C) Partitioning of non-recruited vs. recruited molecules into RGG-based droplets. Enrichment index was calculated as the ratio of fluorescence intensity inside to outside the droplets. Enrichment index of 1 corresponds to equal fluorescence inside and outside the droplets. Tandem RGG constructs were used at 6 μM. (A) Rhodamine (0.01 mg/mL) is enriched in RGG-RGG droplets, while rhodamine-labeled dextrans (10 mg/mL) exhibit enrichment inversely proportional to dextran molecular weight (4.4 kDa, 10 kDa, or 70 kDa). Neither GFP nor RFP (1 μM) are strongly enriched. (B) Enrichment indices of GFP and RFP are insensitive to their concentration. (C) A single RGG domain attached to cargo, RFP-RGG, only results in weak enrichment. In contrast, two recruitment strategies result in strong enrichment: i) Covalently attaching two RGG domains to the cargo (i.e. RGG-RFP-RGG), and ii) non-covalent recruitment via high-affinity interaction pairs (i.e. RFP-SZ2 recruited to SZ1-RGG-RGG). Cargo concentration: 1 μM. Error bars represent standard deviation of $n \geq 3$ replicates. **(D) Cargo release from RGG-based droplets.** Two cargos, GFP-SZ2 and RFP-SZ2 (both with TEV cut sites N-terminal to the SZ2), were co-recruited into SZ1-RGG-RGG droplets and then released upon TEV protease treatment. Release kinetics of both cargos from 5 representative individual droplets are shown.

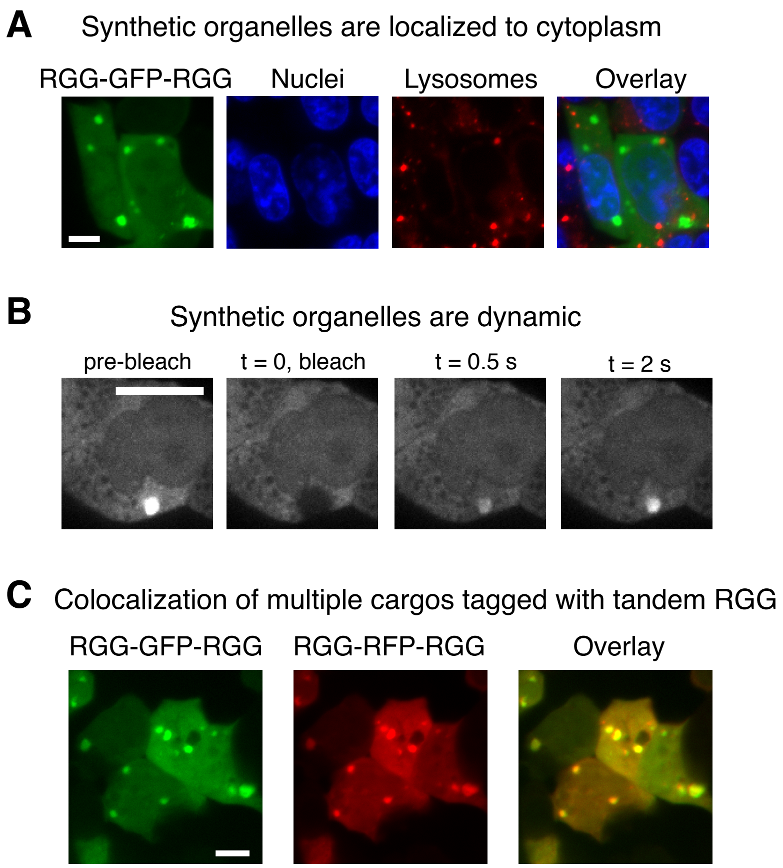

Supplementary Figure 6. RGG-RGG constructs form dynamic synthetic organelles in the cytoplasm of living cells. (A) RGG-GFP-RGG synthetic organelles are cytoplasmic and distinct from other cellular compartments. Live HEK293 cells transfected with RGG-GFP-RGG (green) were stained with a nuclear marker (blue) and lysosomal marker (red). RGG-GFP-RGG droplets are found outside the nucleus and do not colocalize with lysosomes. (B) Fluorescence recovery after photobleaching of an RGG-GFP-RGG droplet in HEK293, showing rapid recovery with $t_{1/2} \sim 1$ s. (C) HEK293 cells co-transfected with RGG-GFP-RGG and RGG-RFP-RGG form droplets with colocalized red and green fluorescence, demonstrating recruitment of multiple cargos by tagging the cargo with two RGG domains. Scale bars: 10 μm.
