# Supplementary material for: Controllable protein phase separation and modular recruitment to form responsive membraneless organelles
Source: Nat Commun. 2018 Jul 30;9:2985. doi: 10.1038/s41467-018-05403-1 (PMC6065366; doi:10.1038/s41467-018-05403-1)
Supplement: Supplementary file 2 — Description of Additional Supplementary Files [file 41467_2018_5403_MOESM2_ESM.pdf]

Description of Additional Supplementary Files

File Name: Supplementary Data 1

Description: Plasmid files in GenBank format

Note: Vectors used for bacterial expression have the prefix pET; vectors for mammalian expression have the prefix pcDNA. For brevity and clarity, the plasmid file name only lists protease cut sites when necessary to distinguish otherwise identical plasmids.
